# Collectanea Medica, Consisting of Anecdotes, Facts, Extracts, Illustrations, Queries, Suggestions, &c.

**Published:** 1817-08

**Authors:** 


### COLLECTANEA MEDICA,

CONSISTING OF

ANECDOTES, FACTS, EXTRACTS, ILLUSTRATIONS, QUERIES, SUGGESTIONS, &c.

RELATING TO THE

*History or the Art of Medicine, and the Auxiliary Sciences.*

Quicquid agunt medici,
nostri farrago libelli.

### *Biographical Anecdotes of the late Mr. Hunter, from the Memoirs published by* Dr. Adams.

[This work being already out of print, may admit of longer extracts than we could otherwise offer.]

"TO render the history of any life interesting, we expect, besides the most scrupulous attention to truth, certain qualities in the writer; viz. a style flowing and suited to the subject; a sufficient knowledge of the character described, with a lively interest for his posthumous fame. It is hoped, that mediocrity in the first may be compensated by an undisputed portion of the latter, as it is scarcely possible to hear an incident or a character described, without, in some measure, participating in the feelings of the narrator.

"Such must be the apology for offering a life which has already been written by an enemy and by a friend, a relation and disciple; besides having appeared in various compilations of Biographies, Dictionaries, and Encyclopædias. But the only one entitled to any credit, was written at an early period after the writer and the world had sustained so heavy a loss. At such a time, the relation of many events might appear less important from their supposed notoriety; nor was it possible to calculate how new and how interesting they might prove to the rising generation. Moreover, there are so many errors, even in the dates of Sir Everard Home, as can only be apologized for by the haste with which the Memoirs are put together. Let me plead lastly, that a new edition of Mr. Hunter's great work has appeared, without a republication of his life.

"Twenty years have passed since we have lamented his loss. From that time, instead of being forgotten, we find his fame gradually increasing. It is already disputed what were his opinions, with a warmth which evinces their importance, and how much they add, by their authority, to the doctrines of others. To what are we to impute this increasing posthumous fame? Not, surely, to those labours which he left unfinished; few of them have appeared, and those few have not always tended to increase our opinion of his perspicuity, whatever they may do of his diligence. His reputation has grown like that of other original geniuses. In proportion as we have since improved in our knowledge of Nature, we see the force of, because we understand, what he taught. In other words, when we make a discovery in pathology, we only learn what we have overlooked in his writings, or forgotten in his lectures."

Some particulars follow relative to Mr. Hunter's ancestry, and the other branches of his family, after which we have an account of

*Mr. Hunter's Education.*

"John Hunter being the youngest child, and born when his father was nearly arrived at his 70th year, was very likely to be a favourite with the whole family. Ten years after his birth, his mother was left a widow. He was now the only son at home, and one or both her surviving daughters were married. It cannot be questioned, that, at this age, he must have been, on all other accounts, extremely interesting. Who shall say that this interest was not increased by the very sound of his name, as associated with the recollection of her late husband at an early period of life. The vigour of his judgment, and the benevolence of his disposition, must have appeared in a thousand juvenile observations.

"Few readers are ignorant that each parish in Scotland is furnished with a grammar-school. These seminaries, fortunately, are not like many of our foundations in England, supported by a specific annuity, which, from the diminished value of money, is scarcely sufficient to afford the school-master a daily breakfast. In Scotland they are usually under the government of the nearest police, and supported by those who have an interest in their good management: add to this, the Scotch are generally the best grammar-masters. I speak it to their honour, and with heartfelt gratitude, that they are usually conscientious in fulfilling this most sacred duty. But the necessary severity and uniformity of life in a grammar-school are not calculated for every genius. Thus we find some of the men who have most distinguished themselves in after-life, have been the most impatient under scholastic restraint. This is not intended as an apology, but as a friendly caution, should these pages accidentally meet the eye of a school-boy. Irksome as the restraint may be, and dull as the employment of construing may seem, they are absolutely necessary for acquiring what can only be acquired at that age; and the want of which becomes, as we advance in life, a perpetual source of regret. Had Captain Cook submitted to this early toil, his writing would have had the charms of polish, as well as the dignity of truth, to recommend them. Had Howard received the education to which his paternal inheritance entitled him, what graces might have adorned the memoirs which even now are a national boast! Had Horne Tooke been sooner aware of the importance of words, he would not have wanted a Wakefield, to complete a work which may form a new æra in philosophy! Had Mr. Hunter availed himself less of maternal tenderness, his style might have been perspicuous, easy, and full of force, without losing any of its accuracy. It is true, in the instance of Mr. Tooke, and still more of Dean Swift, we find that subsequent application may supply the want of early diligence; but this can seldom happen to those whose occupations are constantly and necessarily connected with the busy scenes of life.

" The partiality of a father to a child born in his 70th year, and afterwards the indulgence of a widowed mother to the only remaining boy at home, were not likely to sharpen the industry of John.

Τον δ᾽ ουτι πατηρ ειασκε μαχεσθαι
Ουνεκα οι μετα τοισι νεωτατος εσκε γονοισ
Και οι φιλτατος εσκε.

" Hence, we are not surprized to learn, that, from the period John was sent to school to his seventeenth year, his life passed without any improvement from education. At this time an event occurred, which is only important as connected with such a character.

" There is a general report, that Mr. Hunter was destined to be a carpenter; and one of his biographers goes so far as to say, 'a wheelwright or a carpenter he certainly was.' Had he really been a wheelwright, a carpenter, and the son of a carpenter, his discoveries would not be less brilliant, nor his industry less meritorious.— Sir Everard Home tells us, that 'about this time Mr. Buchanan, who had lately come from London to settle at Glasgow as a cabinet-maker, paid his addresses to Mr. Hunter's sister Janet; and, having many agreeable qualities, she was induced to marry him, although contrary contrary to the advice of her relations. This marriage gave the family great concern; for the qualities which had rendered Mr. Buchanan agreeable, led him into dissipation, and made him neglect his business. Mr. John Hunter, who was now seventeen, went to Glasgow upon a visit to his sister, for whom he had the greatest affection, to comfort her in her distress, and endeavour to assist her husband in extricating himself from his difficulties; but finding, after some time, all his efforts ineffectual, he returned to Long Calderwood.'

"When a youth of seventeen is sent to comfort his married sister, and to extricate her husband, it seems to follow, that he was to take some active share in the business. Even if this was no part of the original intention, those who were acquainted with John's temper, will hardly believe that he could keep from the glue-pot, when orders where pressing, and when the completion of them promised the means of relieving difficulties. In short, there is no doubt, as far as probability and local tradition may be admitted, that John worked at his brother-in-law's trade."

### *Apology for Mr. Hunter's not cultivating Classical Knowledge in After-Life.*

"Mr. Hunter was too honest to devote his valuable time to the gaudy trappings of science. Had he cultivated school-learning at the proper age, he might have written with more confidence, fluency, and perspicuity, but his reasoning could not have been closer, nor his descriptions more correct. In searching for records, we think it right to examine the earliest. In pathology, these are contained in the book of Nature, our researches into which are assisted by the labours of our predecessors. What then must have been the penetration of that man, who in the study of nature has brought into notice passages from writers he was unable to read, and which had been overlooked by profound scholars! By thus resorting to the original source, he collected honey without robbing the hive, or encumbering himself with the wax. Of this I shall at present offer a single example, and also an instance in which he has shown a correctness in the choice of words, which could only be derived from a correct mode of thinking.

"To prove that he discovered in nature what had been overlooked by profound scholars, though accurately remarked by the ancients, it will be surely enough to produce a passage from a scholar and a teacher of medicine, by whom Mr. Hunter is accused of plagiarism from the Greek writers.

"'That people were disposed,' says the learned Dr. Crichton, 'to certain diseases from birth, as well as from the operation of accidental causes, was an observation or a mere matter of fact, which was taken notice of by the Greek physicians, who denominated this cause of disease προηγουμένη; but a certain inaccuracy of expression, in regard to predisposition, has introduced itself into the writings of many medical men since these early times, and has induced them to call certain diseases hereditary diseases. This inaccuracy has probably been caused by the constancy with which hereditary disposition tion operates; but it gave occasion to the late Mr. J. Hunter to ridicule the expression, and to assume the old observations of the Greeks as one of his own discoveries.'

"Thus an 'observation, or mere matter of fact, taken notice of by the Greek physicians,' was overlooked by the most profound scholars for nearly two thousand years, and at length revived by Mr. Hunter. Justly as my departed friend is entitled to this compliment, I should almost have suspected my own partiality, had not the fact been confirmed by Dr. Crichton. I would not, however, have the reader to suppose, that, like Dr. Crichton, Mr. Hunter confuses *disposition* with *predisposition;* both of them '*with accidental causes;*' or that he ever uses such an expression as the '*constant operation of hereditary disposition.*' Like all those philosophers who have improved science, he saw the necessity of first attending to accuracy in the use of terms, and with him all the terms thus jumbled by others have an appropriate and well defined meaning. Equally unjust is the accusation, that he 'ridiculed the expressions of other writers.' Habitually correct himself, he gave others credit, as far as possible, for the same accuracy; and when he differs, seems to do it with some expression of doubt on his own part.

"I shall now produce an example of a reformation in our language, in which he may be as fairly accused of plagiarism, from a standard work on etymology, published nearly twelve years after his death, as of 'assuming for his own the expressions of the Greek fathers.'

'The term coagulable lymph has been adopted by all the later physiologists, whatever may have been their opinion concerning the properties of the blood. Mr. Hunter's objection to it is so pointed, that it might serve as a grammatical example.—' The term coagulable,' says he, 'is more properly applied to substances which might be made to coagulate (are coagulable) only by chemical means.—Perhaps,' continues he, '*coagulating* might be better applied to what is usually called coagulable lymph, and the epithet coagulable might be reserved for those fluids which require a chemical process to produce that effect.'

"Mr. Horne Tooke calls these terminations in *able* the potential passive participle. Nothing can be more exact than Mr. Hunter's illustration in restraining the word 'coagulable to substances requiring a chemical process to produce that effect.' Potential, inasmuch as it is a condition in which they *may* be found; Passive, inasmuch as they have not the power of assuming that condition, but only *suffer* such a change by force; and a Participle, inasmuch as the abbreviation, or winged word, is most conveniently added to a verb.*

"It may be urged, that usage has long ceased to confine this termination to a passive signification. Though this apology may be

"* Treatise on the Blood, p. 16, edit. 1794; Diversions of Purley, Vol. II. chap. viii. anno 1805.

admitted admitted in looser compositions, it is altogether inconsistent with philosophic correctness. As the above etymologist remarks 'To say that the same word was used with two almost opposite ideas, viz. To feel, and To be felt; To beat, and To be beaten; would be just as rational as to say, that the same word should be purposely employed in speech, to signify the horse which is ridden, and the man who rides him.'

"This is only one of numerous instances which might be produced, and many of which have been produced,* in which Mr. Hunter's accuracy of thinking induced, as it always must, a corresponding accuracy of language. The student, who feels dissatisfied with undefined expressions, can alone estimate the importance of such accuracy; and to him it will become familiar, with only the trouble of re-perusing the above passages.

"These two examples are enough to shew, that if his language was not always strictly grammatical, it was perspicuous to those who knew the value of his facts, and were willing to explore them; and that he was much better employed than in studying the classics."

*The Subject continued in the Appendix, with Remarks on the Decline of Mr. Hunter's School under Sir Everard Home, though a King's Scholar at Westminster, and "elected off to Trinity College, Cambridge."*

"We have seen, that when Mr. Hunter made a discovery, in which he had been anticipated by his contemporary, he readily relinquished his claim. In reviving the forgotten discoveries of the ancients, he might have supported his observation with all the triumph of remote antiquity and learned lore; for the coincidence of his remarks with those of the Greek physicians has been already noticed. I shall offer only a few other instances, which, being contained in his lectures, are not accessible to every reader.

"Whilst every Register of Medicines was crowded with cases of lock-jaw, and with successful modes of treatment, no one that I know of, between Celsus and Hunter, paid sufficient attention to one prognostic; yet without attending to this, it is impossible to ascertain what is due to the various and contradictory remedies, and what to the progress of the disease. Mr. Hunter, in his lectures remarked, that, if the spasm is confined to the neck, and the patient survives the fourth day, a favorable prognostic may be formed. That, if it extends beyond the muscles of the neck, and the patient survives the fourteenth day, a favorable change usually commences at that time. Celsus remarks, 'Ea sæpe intra quartum diem tolunt; si hunc evaserunt sine periculo sunt.'† In this he is less guided by the Greek physicians than usual, probably trusting to his own observation in Rome, where the disease might be less fre-

"* See the Appendix: also Introduction to 'Morbid Poisons.

"† Lib. IV. cap. iii. line 5, page 42, ed. Almaloven.

quent, quent than in the more southern parts of the Mediterranean. He is, however, I believe, perfectly correct where the spasm is confined to the neck. Where it extends further, Mr. Hunter is supported by Hippocrates in the prognosis, though the latter protracts the critical days.* From the ancient authors it would be in vain to expect an account of the immediate cause of death, because in their time morbid anatomy was so little cultivated. Mr. Hunter has supplied this desideratum; and his observation, twenty years after his death, has been confirmed by a writer,† unacquainted with him or his opinions on this subject.

"The following is an instance not less to the point:

"Dr. Baillie, with equal modesty and a sense of justice, has the following note in all, excepting the first edition, of his 'Morbid Anatomy.'

"'This diseased change I formerly confounded with the scirrhous enlargement of the uterus, considering them as varieties of the same disease, and therefore blending their description together; but, in consequence of the accurate observations of Dr. Adams, in his 'Treatise on Morbid Poisons,' I have thought it proper to separate them.'—*Morbid Anatomy, Second Edition; Article, Ulcer of the Uterus.*

"Though Dr. Baillie has honoured me so far as to give me credit for the above observation, it becomes me to acknowledge the source from which I derived it; and it is with no small satisfaction I have since seen the same sentiment expressed by Aretæas, with scarcely any difference but the language of the two;‡ so that had Mr. Hunter understood Greek, he might have been accused of plagiarism; an accusation which cannot be brought against any scholar who has written on cancer.

"The two diseases above referred to may, with much greater propriety, be called the opprobria chirurgorum than the gout opprobrium medicorum. Not to be able to cure a disease may be pardonable; but nothing can excuse the slightest inattention to its phenomena, particularly to those characters by which it is to be distinguished. It is but justice to the rising generation, to admit a progressive improvement in all these nicer distinctions; and if many of them are not yet aware of the sources from which they derive such advantages, Mr. Hunter, in this respect, only shares the fate of most others who have improved science. He may, indeed, be considered more fortunate than his predecessors, inasmuch as what was entirely overlooked in them is slowly attracting notice when repeated by him.

"Happy had it been for the four last centuries if another passage in Celsus had been earlier attended to. Is Mr. Hunter again to be

---

"* Hippocr. sect. v. p. 125, ed. Foesii.

"† By the spasm extending to the heart. See a valuable paper by Mr. Howship, Lond. Med. Journal, vol. xxii. page 188.

"‡ Aretæus de Caus. Morb. Diuturn, lib. ii. chap. 12."

accused of plagiarism from the ancients, because he almost translates a whole chapter, which, though immediately connected with the most learned and widely-extended controversy in medicine, has been in its application overlooked by all the learned? The medical reader will at once refer this to the chapter in Celsus, *De obscœnarum partium vitiis*, in which we meet with an accurate description of every kind of ulcer on those parts first distinguished by Mr. Hunter from the truly syphilitic. How gladly would he have availed himself of such an authority we may easily conceive, by the reference he makes to so many modern writers.

"What has been just remarked, is not to be considered as a mere critical inquiry. The suggestion of Mr. Hunter gave rise to the first attempt at distinguishing the various diseases at one time indiscriminately confounded under the term cancer; and it is now frequently remarked, that, by reviving one opinion from Aretæus, he has saved many a saint from an operation, and, by reviving another from Celsus, many a sinner from repeated salivations.

"In considering the above, we cannot but admit that medicine is less progressive than most other sciences. It may be urged, indeed, that important facts, though noticed by the ancients, are mixed with fables, and preserved in a language little cultivated by those who are most engaged in practice. The obvious inference is, that our records should be confined to well ascertained facts, and that those facts should always be kept in view. On this account, I have often felt surprised, that Sir Everard Home should regret the caution, or, as he calls it, the tardiness of Mr. Hunter, in giving his observations to the public. 'But (continues the same writer) such was his turn for investigation, and so extensive the scale upon which he instituted his inquiries, that he always found something more to be accomplished, and was unwilling to publish any thing which appeared to himself unfinished. His Observations on the Muscular Action of the Blood-vessels were laid before the Royal Society in 1780, and yet he delayed publishing them till his Observations on the Blood and Inflammation were arranged, and they will be found to make a part of the present work.'

"After these remarks, we may still hope to see some valuable fragments, at least, from so desirable a source. Whenever they appear, it is much to be wished that they may be in the author's own words, however unconnected they may seem. The 84th vol. of the Philosophical Transactions, published a year after his death, contains, 'Facts relative to Mr. Hunter's preparation for the Croonian lecture, by Everard Home, Esq.' In the subsequent part of the same volume, the fallacy of these 'facts' is shown by the person who brought them forward. New 'facts' are introduced, confirmed by experiments, illustrated by plates, witnessed by Mr. Ramsay; and among these facts, we are informed, that the eye loses none of its adjusting powers by a deprivation of the lens. In the succeeding volume, these 'experiments' are shown to be uncertain. All this may evince candour, and may be considered as a tribute to the memory of the deceased, by thus bringing his successor into notice.

But we cannot, in the midst of it, fail to applaud the prudence of Mr. Hunter, who, whatever his opinions were, knew better than to consider them as 'facts,' or to offer them in their crude state to the public.

"Since the days of Bacon, we have learned to be satisfied with nothing less than facts. Hence, an experiment is often considered as necessary in the support of every theory; and we are apt to suppose, that every experiment is worth being recorded. Men accustomed only to the changes which they detect in common matter, are seldom aware of the intricacy of experiments, when made on living matter. The bare making them on living animals induces a new train of actions, or the cessation of certain actions, which the inexperienced operator is apt to confound with the result of his inquiry. Another maxim should be always kept in view, that when a fact is once proved, all experiments to confirm it are unnecessary.

"I was led to these considerations by Mr. Brodie's paper, in the Philosophical Transactions, 'respecting the influence of the brain on the actions of the heart, and on the generation of animal heat.'

"'In making experiments (says this gentleman) on animals, to ascertain how far the influence of the brain is necessary to the action of the heart, I found, that when an animal was pithed by dividing the spinal marrow in the upper part of the neck, respiration was immediately destroyed; but the heart still continued to contract circulating dark-coloured blood, and that, in some instances, from ten to fifteen minutes elapsed before its action had entirely ceased. I further found, that, when the head was removed, the divided blood-vessels being secured by a ligature, the circulation still continued, apparently unaffected by the entire separation of the brain. These experiments confirmed the observations of Mr. Cruikshank and M. Bichat, that the brain is not directly necessary to the action of the heart, and that when the functions of the brain are destroyed, the circulation ceases only in consequence of the suspension of respiration. This led me to conclude, that, if respiration was produced artificially, the heart would continue to contract for a still longer period of time after the removal of the brain. The truth of this conclusion was ascertained by the following experiments.'

"Eight or nine experiments follow, made on living animals; the result of which is, that, by keeping up artificial breathing, after the heart is deprived of all connexion with the brain, the circulation may still be continued; but the secretions and animal heat no longer continue. From this, Mr. Brodie concludes, that the action of the heart is independent of the brain; that animal heat does not depend on the circulation, but on the nervous system; and that the secretions cease when deprived of the nervous influence.

"Respecting the first, (that the brain is not directly necessary to the action of the heart) though only Cruikshank and Bichat are mentioned, yet Mr. Brodie's experiment in proof of it, was only a repetition of Mr. Hunter's, and by probably the same instrument.—'I invented (says Mr. Hunter) a double pair of bellows, constructed in such a manner as, by one action, to throw fresh air into the

 lungs; lungs; and by another, to *suck out again* the air which had been thrown in by the former, without mixing them.'*—A small tube (says Mr. Brodie) of elastic gum was so connected to a pair of bellows, that the lungs might be inflated and allowed to empty themselves.' By these contrivances, whether the same or not, each proved the same thing, and no more; namely, that the action of the heart seems sympathetic with respiration. Both continued their experiments about the same time. Mr. Brodie observed further, that, 'in such a state of things the secretions were discontinued, and the generation of heat also.'

"Did it prove, then, that heat is independent of the circulation; because, under such a circulation, and thus mutilated, the animal grew colder? or, did it prove that the brain and nerves were alone necessary for the generation of heat; because, when the head was separated, no heat was generated? I mean not by this to undervalue Mr. Brodie's talents. I have no doubt that, at this time, he would gladly recall his papers. But I cannot help urging any future young experimenter, that he should learn what such men as Mr. Hunter have done on the subject of their inquiries, and also recollect his remark on the necessity of frequent experiments before we trust to the result of any other. Had Mr. Brodie done this, he would have found, that, though Mr. Hunter had anticipated his experiment, he was too prudent to form such hasty conclusions. To show that animal heat is not entirely dependant on circulation, he took the opportunity of an apoplectic subject, whose external heat was preserved by the bed-clothes, and whose pulse was regular; yet, even here, he found the heat vary. This might have led him to suppose, that animal heat depends on the brain, as that organ was oppressed; but numberless arguments occurred to convince him of the contrary; and he concludes by observing, that animal heat, like the secretions, depends on the powers of the whole system, and that, in proportion as they are more perfect, the standard heat will be more perfectly preserved.†"

The case follows, extracted from Mr. Abernethy's Essays, after which the author remarks, that, in most cases in which there is any permanent obstacle to the transmission of the blood through the lungs, there seems a difficulty in preserving the standard heat.

---

"* Treatise on Blood, p. 152. Animal Economy, second edition, page 134."

"† Anim. Œconomy, p. 103 and 104. It is worth while to observe how nearly this language coincides with Celsus—Neque enim natura sanguinis est ut calet, sed ex his quæ in homine sunt, is vel calescit vel frigescit, lib. iv. cap. 3.—Since writing the above, the second edition of Blumenbach's Physiology has reached me; in which I see he makes no scruple to give the old doctrine of the chemical production of animal heat. This I should not have attended to

*Anecdotes of the late Dr. Lettsom; extracted from Memoirs of his Life and Writings*, by T. J. PETTIGREW, Esq.

"JOHN COAKLEY LETTSOM was one of a twin, born at Little Vandyke, on the 22d of November 1744.* At six years of age he departed from the West Indies.

"About two miles from Lancaster, near which was a school under the direction of Gilbert Thompson, an unmarried man, whose house was kept by his sister. He was celebrated among the Society of Friends. Under his tuition Lettsom was placed, and lodged at the house of Mrs. Barnes, then a widow. The affectionate Lindo, the faithful friend of Lettsom's father, took him to this rural residence. The house was situated in the parish of Sankey, which gave name to a street in Warrington. Within about 200 yards of this residence ran a stream of water, or brook, which empties itself into the river Ribble, and divides the parish of Sankey from Penketh, in which parish the school was erected. Adjoining to it was a meeting-house for the Society, which was frequently visited by Samuel Fothergill. A common, or heath, about three miles in circumference, intervened between the dwelling-house and the school. The number of boys at the school varied from 40 to 60: they were governed and instructed by the master and an usher, his nephew, of the same name. These particulars are here introduced, because they materially contributed to the preservation of his health, and the invigoration of his constitution.

"The widow Barnes had a son settled in Liverpool, to whose house she retired, and took Lettsom with her.

"His removal to Liverpool took place about the conclusion of his 14th year. The death of his father had been recently communicated to him; and soon afterwards he heard of the second marriage of his mother to Mr. Samuel Taine, and that his father's executor had neglected his property, and had disposed of the sugar-plantation in Cane Garden Bay. No instructions had been recently transmitted

---

to, but for a note which revived the recollection of one of my own remarks about twenty-five years ago.

"Blumenbach's note is as follows:—'Hence the constant coldness of those wretched beings who labour under the blue disease, which arises from a mal-conformation of the heart. Sometimes the septa of the heart are imperfect, sometimes the aorta arises from the right ventricle, as in the tortoise. Among innumerable instances of this lamentable disease, suffice it to quote John Abernethy's Surgical and Physiological Essays, part iii. p. 158; and Fr. Tredman's Zoology, t. i. page 177.'"

"* The Doctor informed the writer of this, that his mother had seven times had twins, all of whom were males. He and his twin brother Edward were the last children borne by her, and the only two who survived."

to to direct his studies; it was, however, at length concluded, that he should attend a seminary in Liverpool, to learn merchant's accounts, preparatory to admission into a mercantile house. This plan was pursued until he had reached his 15th year, when an event occurred which diverted him to another pursuit.

"He had a distant relation in Tortola, named John Pickering, whose two youngest sons, Isaac and Josiah, by his last wife, were sent to the care of Samuel Fothergill, who placed them at Penketh school; but, on its dissolution, they were removed to a school at Shelborne.

"John Pickering had by his first wife a son, named after himself, who, on his return to Tortola, married the daughter of Bezaliel Hodge, of the same island. John Pickering visited England for curiosity and improvement. On his arrival at Liverpool, he was met by Samuel Fothergill, who always appeared to feel great interest in promoting Lettsom's happiness; and he proposed to him to join in his guardianship. This being settled, it was concluded to send him apprentice to Abraham Sutcliff, a surgeon and apothecary at Settle in Yorkshire. Accordingly, a pack-horse that used to pass from Lancashire to Yorkshire was hired, and upon it Lettsom travelled to Settle. This was in April 1761. The little classical knowledge he had attained at Penketh was dissipated during the time he spent in acquiring the mercantile routine. Thus prepared, he was to be qualified for the most important professional duties, and that too in an obscure part of the country. Mr. Sutcliff was distantly related to S. Fothergill, at that time Lettsom's guardian, and who, no doubt, entertained a favourable opinion of his new tutor, who proved, happily for Lettsom, a man of no ordinary talents. Sutcliff was an excellent classic, although self-taught; and so attached was he to Latin, that he never would permit Lettsom to read an English book in his presence, and often would instruct him in his favourite language.

"When Lettsom arrived, Sutcliff possessed an amiable wife, the mother of two children. Her condescension and kindness added much to his comfort, during the whole of his residence at Settle.*

"Dr. Lettsom was ever exceedingly grateful to Abraham Sutcliff for the attentions he received from him. The latter having, in 1780, accumulated by professional industry a sum of money (about 10,000l.) was anxious to retire from the more arduous parts of professional exertion in favour of his son. Dr. Lettsom's flourishing condition in London at this period did not obliterate the esteem and gratitude so justly due to Sutcliff. He was urged and even offered 100l. by the Doctor, to pay his expences in coming to London to visit his old apprentice. It was not till after the lapse of several years that Mr. Sutcliff paid this visit, which continued fourteen days. Against his arrival Lettsom had procured for him a diploma of

"* Sutcliff received a fortune of 500l. by his marriage. He had eight children, some of whom are at this time living."

Doctor

Doctor of Medicine. He addressed him by observing, that he was now old, that he had a son qualified for his profession, and that he ought to retire and enjoy the easier earnings of a physician. Sutcliff replied, that he had designed it, but was at a loss to apply for a diploma, as his old teachers were no more. Dr. Lettsom presented one to him. How surprised and delighted was the good old man to find that he was already one of the diplomatic body: 'My lad,' said he, with his eyes suffused with tears of joy, 'this is more than I know how to acknowledge!'

"About the second year of Lettsom's apprenticeship, he began to visit patients, when his master was out of town or engaged by midwifery; but, never having heard a lecture, nor seen any anatomical figure, except a skeleton, he could but be ill qualified to discharge the functions with which he was intrusted. He had, however, read much, and had, by that means alone, acquired some knowledge of comparative anatomy. In Sutcliff's library he was assisted by Keil's Anatomy, Monro on the Bones, Douglas on the Muscles, and Winslow's Anatomy. In medicine, were some of Boerhaave's works, and Shaw's Practice of Physic: a very incompetent medical library, it must be acknowledged!

"Lettsom was so early sensible of the want of a good memory, that at this time (being in his 18th year) he availed himself of notes, and constructed tables, to assist it; and by often reverting to them, the impressions that he wished more particularly to retain, were rendered so strong as rarely to elude recollection. Thus, with moderate powers of mind, he was enabled to supply, by industry and art, what nature had denied him. By the construction of tables, he surmounted many difficulties which occurred in the course of his attention to anatomy, and was thus prepared the better to understand what he had collected by reading.

"By the attention to his master, aided by his own application, he recovered his knowledge of the Latin language, and was then anxious to acquire such a proficiency in the French tongue, as would qualify him to read an author. To effect this, a party of his acquaintance, of both sexes, united in expence to procure a French master from London; and, by due application, in three months they could read the language with facility, and both speak and write it so as to be understood. Soon afterwards Lettsom procured a few Greek books, and, with Stackhouse's Grammar, acquired a little knowledge of this language also; but he never could overcome the early neglect of his classical education.

"His most favourite study, indeed, was botany. To assist him in it he borrowed Gerard's Herbal; and in his excursions in the vicinity of Settle, he collected many good specimens of rare plants, with which he composed an Hortus Siccus. From other specimens he made impressions on paper, which resembled drawings, and may be done with very little trouble.

"After having completed the five years' apprenticeship in Settle, under the superintendance of Sutcliff, Lettsom determined to emerge from a small market-town, to enter the metropolis of England, without without having a relation, or knowing a friend in it. Many worthy people in Settle, whose good opinion he had secured, advised him against his intended journey, in consequence of four apprentices from the same house having died soon after their entrance into London. Lettsom, however, was not to be diverted from his intention; and his guardian, Samuel Fothergill, seeing no obstacles, recommended him to his brother, Dr. John Fothergill, one of the most celebrated physicians in Europe. Lettsom arrived in London, and waited upon him in the summer of 1766.

"The distance of his apartment in Gracechurch-street was convenient for attendance at St. Thomas's Hospital, where he entered as a surgeon's dresser, under Benjamin Cowell, Esq. The other surgeons were Mr. Baker and Mr. Smith, men of no great eminence. The physicians were Akenside, Russell, and Grieve. Lettsom was early fond of poetry, and had read the 'Pleasures of Imagination' with admiration. He anticipated great pleasure in coming under the author's notice; for, by a small premium, a surgeon's pupil is admitted to the practice of the physicians of the Hospital.

"Great, however, was his disappointment, in finding Dr. Akenside the most supercilious and unfeeling physician that he had hitherto known. If the poor affrighted patients did not return a direct answer to his queries, he would often instantly discharge them from the Hospital. He evinced a particular disgust to females, and generally treated them with harshness. It was stated that this moroseness was occasioned by disappointment in love; but hapless must have been that female who should have been placed under his tyranny. Lettsom was inexpressibly shocked at an instance of Dr. Akenside's inhumanity, exercised towards a patient in Abraham's Ward, to whom he had ordered bark in bolusses; who, in consequence of not being able to swallow them, so irritated Akenside, as to order the sister of the Ward to discharge him from the Hospital; adding, 'he shall not die under my care.' As the sister was removing him, in obedience to the Doctor, the patient expired.

"One leg of Dr. Akenside was considerably shorter than the other, which was, in some measure, remedied by the aid of a false heel. He had a pale strumous countenance, but was always very neat and elegant in his dress. He wore a large white wig, and carried a long sword. Lettsom never knew him to spit, nor would he suffer any pupil to spit in his presence. One of them once accidentally did so, yet standing at some distance behind him. The Doctor instantly spun round on his artificial heel, and hastily demanded, who was the person that spit in his face? Sometimes he would order some of the patients, on his visiting days, to precede him with brooms to clear the way, and prevent the patients from too nearly approaching him. On one of these occasions, Richard Chester, one of the governors, upbraided him for his cruel behaviour: 'Know,' said he, 'thou art a servant of this Charity.'

"On one occasion his anger was excited to a very high pitch, by the answer which Mr. Baker, the surgeon, gave to a question the Doctor put to him, respecting one of his sons, who was subject to epilepsy, epilepsy, which had somewhat impaired his understanding. 'To what study do you purpose to place him?' said Akenside to Baker. 'I find,' replied Baker, 'he is not capable of making a surgeon, so I have sent him to Edinburgh to make a physician of him.' Akenside turned round from Baker with impetuosity, and would not speak to him for a considerable time afterwards.

"Dr. Russell was as condescending, as Akenside was petulant. Akenside, however, would sometimes condescend to explain a case of disease to the pupils, which always appeared sagacious; and, notwithstanding his irritable temper, he was more followed than Russell by the pupils. Dr. Russell resided at No. 1, Church-court, Walbrook.

"Dr. Grieve lived in the Charter House, to which he was physician. He was an amiable man, and an unassuming scholar. He was the translator of Sydenham.

"The pecuniary circumstances of Lettsom did not enable him to continue longer than twelve months in London, and excluded him from the school of Edinburgh. He, accordingly, devoted his time incessantly to the Hospital, and the lectures which London afforded. He was a constant attendant of the physicians in their walks through the wards, and embraced as many opportunities as he possibly could to avail himself of the advantages arising from it. In the morning, early, before any attendance was given, he usually visited many select patients, wrote out the symptoms, and afterwards examined the prescriptions of the physicians. On some particular cases, he compared the writings and practice of authors, and gradually acquired a considerable degree of precision in anticipating both the practice and the remarks of the faculty. If he thought himself much at a loss, he repeated his visits to the hospital in the afternoon; and this not only confirmed the little knowledge he had acquired, but likewise gave him a frankness and ease of demeanour at the bed-side. During all his attendance, however, no other pupil adopted this system, which he found so highly interesting to his advancement. At the same time, he continued to take notes of, and make reflections upon, what he saw; and thus acquired a method of investigation and decision, which ever afterwards proved of the highest use in determining his medical conduct and practice. Until his return from the West Indies in 1768, he never could claim the advantages of any other lectures than those afforded by a course of midwifery and anatomy. His private clinical practice was a substitute for the rest.

"During Lettsom's attendance at the hospitals, no person was more kind to him than Richard Chester. At the same time that he maintained the orthodox friend, he possessed an affectionate and liberal mind, which led him to view the failings of others with a forgiving disposition. He was very cheerful and pleasant in company, steady in his friendships, and open-handed to the poor.

"The time now arrived when Lettsom was to bid adieu to those few, but highly-valued, acquaintances. In the interviews he had with Dr. Fothergill, and the opportunities of witnessing his wonderful routine of professional employment, he could not avoid feeling an ambition to settle in the metropolis. There seemed, however, to be an insurmountable obstacle opposed to such an event, in his pecuniary situation, and in the wide distance from Europe of his native island.

"On the 8th of December he arrived at Tortola, and remained there until July 1768.

"Lettsom's object in returning to his native island was to take possession of the little property left him by his father, which then consisted of a small portion of land, and about fifty slaves. At this time he was not possessed of 50l. in the world; but, viewing the traffic in living blood as wicked and unlawful, he immediately *emancipated* them, and became a voluntary beggar at the age of 23. He never repented of this sacrifice; indeed, Heaven soon repaid it, by conferring upon him innumerable temporal blessings; and, what must be estimated still more highly, a grateful heart to diffuse them among his poorer fellow creatures. He did not liberate his slaves from any advice of the Society to which he belonged—he did not do it from religious motives, merely as such, but he had early read much; he had considered the tenets of different religions and professions, and he thought there was only one true religion, consisting of doing unto others as we wish they should do unto us. A demure face, and all the sanctimonious exteriors of individuals, he apprehended as nothing, where beneficence was wanting. Impressed with these sentiments, as he lived, so he died. But he lived to correct his opinion as to the mode by which the emancipation of slaves should be effected. In a letter to the Rev. Dr. Madison of Virginia, dated September 24, 1804, he observes, 'There is one subject which I have slightly hinted at, the toleration of slavery in America; and, after perusing the excellent pamphlet on the emancipation of your slaves, with which thou some months ago favouredst me, I feel a difficulty in determining upon any plan adequate to the magnitude and urgency of the object.

"'To sell them to others for future slavery would be the height of cruelty; and to continue them with yourselves, in perpetual slavery, is impolitic and dangerous, as you have recently experienced, as would also be their immediate and complete freedom. I conceive, with every dispassionate writer, that a *gradual emancipation* is *alone* feasible, and, in my opinion, easily practicable. It requires, however, a considerable length of time to teach a slave to be free: therefore, emancipation should be gradual; for were it immediate and general, slavery would degenerate into sloth and unbridled licentiousness. The example of the late Prime Minister, Count Bernstoff, of Denmark, should be adopted. He liberated his slaves with the most scrupulous caution and parental attention. He first allotted to each of them a piece of land, and instructed them to plough, sow, and otherwise cultivate the soil. He obtained for them tools and utensils of every requisite kind, at a considerable expence; and, during this course of instruction, he supported them under every exigency or want, to which such a novel state had introduced them.

In a few years, they not only maintained themselves, but acquired a surplus to pay him a rent, and the old Count lived to see his domains four-fold in value, and the freemen, whom he had reared from a state of slavery, enjoying ten-fold happiness. One of the Count's estates was traversed by four great roads; in the centre where they met, he lived to see a statue erected to himself, as a monument of their gratitude. This was erected solely at their expence.'

"At Tortola, Lettsom commenced practice, and, in the course of five months, succeeded in amassing nearly 2000l. half of which he gave to his mother: with the remainder he returned to London, with the view of following the steps of the great Dr. Fothergill.

"In September 1768, he landed at Liverpool, and after spending a short time with Samuel Fothergill, at Warrington, arrived in London. In October he set off for Edinburgh, and attended Dr. Cullen's clinical lectures, and his lectures on the institutions of medicine. He also attended Dr. F. Home on the materia medica; after which he proceeded to Paris, Spa, Aix-la-Chapelle, and other situations favourable to the obtaining of medical knowledge, or the reception of invalids for the benefit of change of air, the drinking of particular waters, &c. He had obtained from eminent men several letters of introduction to the professors and principal literary characters in these countries, which were of the greatest use. With many of these distinguished persons he maintained a correspondence for many years. Among these were the celebrated Macquer, Le Roy, Vicq d'Azyr, and Dr. Dubourg, to whom he was introduced by a letter from the celebrated Dr. Benjamin Franklin.

"After visiting the different schools, he took his degree of Doctor of Medicine at the University at Leyden, on the 20th of June, 1769. His thesis was entitled, 'Observationes ad vires Theæ pertinentes;' and was inscribed to his patron Dr. John Fothergill, to his guardian Samuel Fothergill, and to his old master Abraham Sutcliff.

"Soon after graduating, he returned to London; and, in 1770, having become a Licentiate of the Royal College of Physicians, he commenced practice under the protection of Dr. Fothergill. In this year also he was elected an Honorary Member of the Physico-Medical Society of Edinburgh.

"Dr. Lettsom was now rapidly rising in his profession, and he determined upon forming a matrimonial connexion with an amiable young lady, the daughter of Mr. Miers, a wealthy tin-plate-worker, resident in Cannon-street. This desirable event took place on the 31st July, 1770: an union formed on the most durable basis, increasing in strength every year of their existence. There could not be a more tender, engaging, or attentive partner than the Doctor, whose perpetual solicitude, the writer of this has repeatedly had the happiness of observing, was particularly directed to the increase of her comforts. She lives to deplore her irreparable loss. By this marriage Dr. Lettsom acquired a very considerable fortune, which enabled him to exercise those acts of beneficence (which had hitherto

R 2 been been in a degree confined) upon a more extended scale. His liberality, however, laid him open, on many occasions, to the impositions of hypocritical knaves, whose deep and dark designs he was unfortunately far from suspecting.

" In this year he was chosen a Fellow of the Society of Antiquaries; and, in the following one, a Fellow of the Royal Society."

A very minute account follows of Dr. Lettsom's practice and writings; after which—

" The most painful part of the author's duty now commences—a recital of the circumstances connected with the dissolution of Dr. Lettsom. For some time he had been attending a gentleman whose case proved fatal, and he was desirous that the body should be examined: this was chiefly performed by the Doctor himself, on the 22d of October, 1815. He remained in a cold room for two hours, and, on the following day, felt chilly and unwell, but not so as to excite much alarm. On the 25th the following letter was transmitted to the author:

" ' DEAR MR. PETTIGREW,

" ' I was attacked yesterday with a severe rigor of fever, which sent me to bed at six o'clock in the afternoon, and I had a dreadful night; but I rose at ten this morning somewhat better, and shall endeavour to visit a few patients, and, as early as I can after my home patients, seek a retreat in bed; so that, to my great regret, I cannot attend the Philosophical Society without danger to the health of Your's sincerely,

" ' *Sambr. Co. Oct.* 26, 1815. J. C. LETTSOM.

" ' For the last twenty-seven years I have not been confined by illness.'

" On the 27th the author visited him, and, alas! found him labouring under a strong rigor—(a severe cold shivering fit,) indicative of approaching fever, and complaining of great soreness of his arms, which he (Dr. Lettsom) considered to be rheumatic. The necessity for great care was immediately urged; and he was requested to see his friend Dr. Babington. He, however, observed that he should be better in a few days, and that he wished for no one to attend him. At that time he had a poor patient resident in Whitecross-street, whom he was determined to visit, against which his friends strongly contended, but fruitlessly. He went out, and returned, literally unable to get out of his carriage, and suffering the most acute pain upon any attempt to be assisted. In the evening he was visited by his friends Dr. Babington and Mr. Norris, and was confined to his room. The next day his disease assumed a more distinct character, and he was unable to move in his bed without assistance, yet sustaining, with the greatest fortitude, the most excruciating pain. In this situation, his anxiety for his patients was unabated—he requested the author to visit them, and was eager to know the progress of their diseases. Perpetual inquiry was directed to to the Philosophical Society, and respecting the arrangements for the approaching Anniversary, concerning which he was so interested that he said, provided he was only able to sit, and not even to speak on that occasion, he would attend it.

"On the 30th he appeared improved, but on the 31st great debility came on, attended with slight delirium, which terminated his valuable existence on Wednesday the 1st of November, between three and four o'clock in the morning. A life like his was calculated to render its close painful to his survivors only; and he died without a groan.

"Intelligence of this melancholy event soon spread throughout the metropolis: hundreds of persons walked up Sambrook-court to view the mansion of its late owner, in order to ascertain the truth of the report. The Medical and Philosophical Societies suspended their meetings until the usual funeral ceremonies were performed. The Monday following his interment, a tribute of respect and gratitude was paid to his memory by the Medical Society, in adopting the following Resolutions:

"'*Resolved*,

"'That the Society receive the account of the decease of their late much-valued associate with feelings of deep regret for his loss; of unfeigned respect for his memory; and of gratitude for the numerous services rendered by him to the Society.

"'That the above Resolution be entered in the minutes, and subscribed by the President; and that a copy be transmitted to his son, Samuel Fothergill Lettsom, Esq.'

"In the subsequent Anniversary Oration in March, by Dr. Clutterbuck, a very appropriate eulogy on his character was introduced, his services to the Society acknowledged, and his loss feelingly and justly lamented.

"The meetings of the Philosophical Society of London were suspended until the 21st of November, which day was appropriated to the delivery of an Eulogy by the writer of this Memoir. This was composed at the particular request of the Council of the Society, and the eulogist selected from among the members in consequence of his intimacy with the worthy Doctor, and his introduction of him to the Society."

CRITICAL

